# Treatment with the nitric oxide synthase inhibitor L-NAME provides a survival advantage in a mouse model of *Kras* mutation-positive, non-small cell lung cancer

**DOI:** 10.18632/oncotarget.9874

**Published:** 2016-06-07

**Authors:** Nicole L.K. Pershing, Chi-Fu J. Yang, MengMeng Xu, Christopher M. Counter

**Affiliations:** ^1^ Department of Pharmacology & Cancer Biology, Duke University School of Medicine, Durham, NC, USA; ^2^ Department of Surgery, Duke University School of Medicine, Durham, NC, USA; ^3^ Department of Radiation Oncology, Duke University School of Medicine, Durham, NC, USA

**Keywords:** nitric oxide synthase, nitric oxide, non-small cell lung cancer, RAS, preclinical trial

## Abstract

Oncogenic mutations in the gene *KRAS* are commonly detected in non-small cell lung cancer (NSCLC). This disease is inherently difficult to treat, and combinations involving platinum-based drugs remain the therapeutic mainstay. In terms of novel, pharmacologically actionable targets, nitric oxide synthases (NOS) have been implicated in the etiology of *KRAS*-driven cancers, including lung cancer, and small molecular weight NOS inhibitors have been developed for the treatment of other diseases. Thus, we evaluated the anti-neoplastic activity of the oral NOS inhibitor L-NAME in a randomized preclinical trial using a genetically engineered mouse model of *Kras* and *p53* mutation-positive NSCLC. We report here that L-NAME decreased lung tumor growth *in vivo,* as assessed by sequential radiological imaging, and provided a survival advantage, perhaps the most difficult clinical parameter to improve upon. Moreover, L-NAME enhanced the therapeutic benefit afforded by carboplatin chemotherapy, provided it was administered as maintenance therapy after carboplatin. Collectively, these results support the clinical evaluation of L-NAME for the treatment of *KRAS* mutation-positive NSCLC.

## INTRODUCTION

Oncogenic mutations in *KRA*S are detected in upwards of a quarter of non-small cell lung cancers (NSCLC), and are associated with resistance to EGFR inhibitors and potentially also other chemotherapeutics [1]. There are no clinical agents to inhibit oncogenic KRAS, and frontline chemotherapy for advanced NSCLC typically combines a platinum-based drug with an anti-mitotic or DNA damaging agent, yielding a median survival between 8 and 11 months [2]. Immune checkpoint inhibitors are showing clinical promise, but to date have a limited overall response rate in NSCLC [3]. As such, there is a need to develop novel therapeutics for the treatment of *KRAS* mutation-positive NSCLC.

Nitric oxide synthase (NOS) enzymes are potential new therapeutic targets in lung cancer. The NOS family, comprised of nNOS (NOS1), iNOS (NOS2), and eNOS (NOS3), convert arginine to nitric oxide (NO) and citrulline [4]. While the role of NOS enzymes in cancer is complex [5], with regards to *KRAS* mutation-positive NSCLC, the murine lung cancer cell line LLC was reported to grow more poorly when implanted into *eNOS^−/−^* mice [6]. *iNOS^−/−^* mice have also been shown to be resistant to *Kras* mutation-positive lung tumorigenesis induced by either the carcinogen urethane [7] or genetic activation of an inducible oncogenic *Kras* allele in the lung [8]. Conversely, ectopic expression of iNOS was shown to increase the tumor growth of the *KRAS* mutation-positive Calu-6 human lung carcinoma cell line [9]. Although *NOS* isoforms are not consistently up-regulated in NSCLC tumor cells [10–14], multiple studies have documented higher levels of exhaled NO from lung cancer patients [10, 11, 15, 16], which has been linked to macrophage infiltration [11]. As such, accumulating evidence points towards a possible role of NOS in NSCLC.

Another feature of the NOS family that makes them attractive as therapeutic targets is that there are a host of small molecules that inhibit these enzymes [4]. Of these, the drug *N*^G^-nitro-L-arginine methyl ester (L-NAME) is one of the most clinically developed pan-NOS inhibitors; evaluated for the treatment of septic and cardiogenic shock [17, 18], as well as in non-disease settings (*e.g.* [19]). L-NAME has also been shown inhibit tumorigenesis in various *in vivo* cancer models [20–23]. Collectively, these observations support the preclinical evaluation of L-NAME for the treatment of *KRAS* mutation-positive NSCLC.

Admittedly, a major challenge in developing a new therapeutics is to accurately predict the anti-neoplastic activity in a preclinical setting [24]. As mice developing NSCLC induced by activation of mutant alleles of *Kras* and *Trp53* recapitulate the therapeutic responses observed in the clinic [25], we chose to evaluate the anti-neoplastic activity of the pan-NOS inhibitor L-NAME in this genetically engineered mouse model of *Kras* mutation-positive NSCLC.

## RESULTS

### L-NAME therapy reduces tumor burden

**Figure 1:**
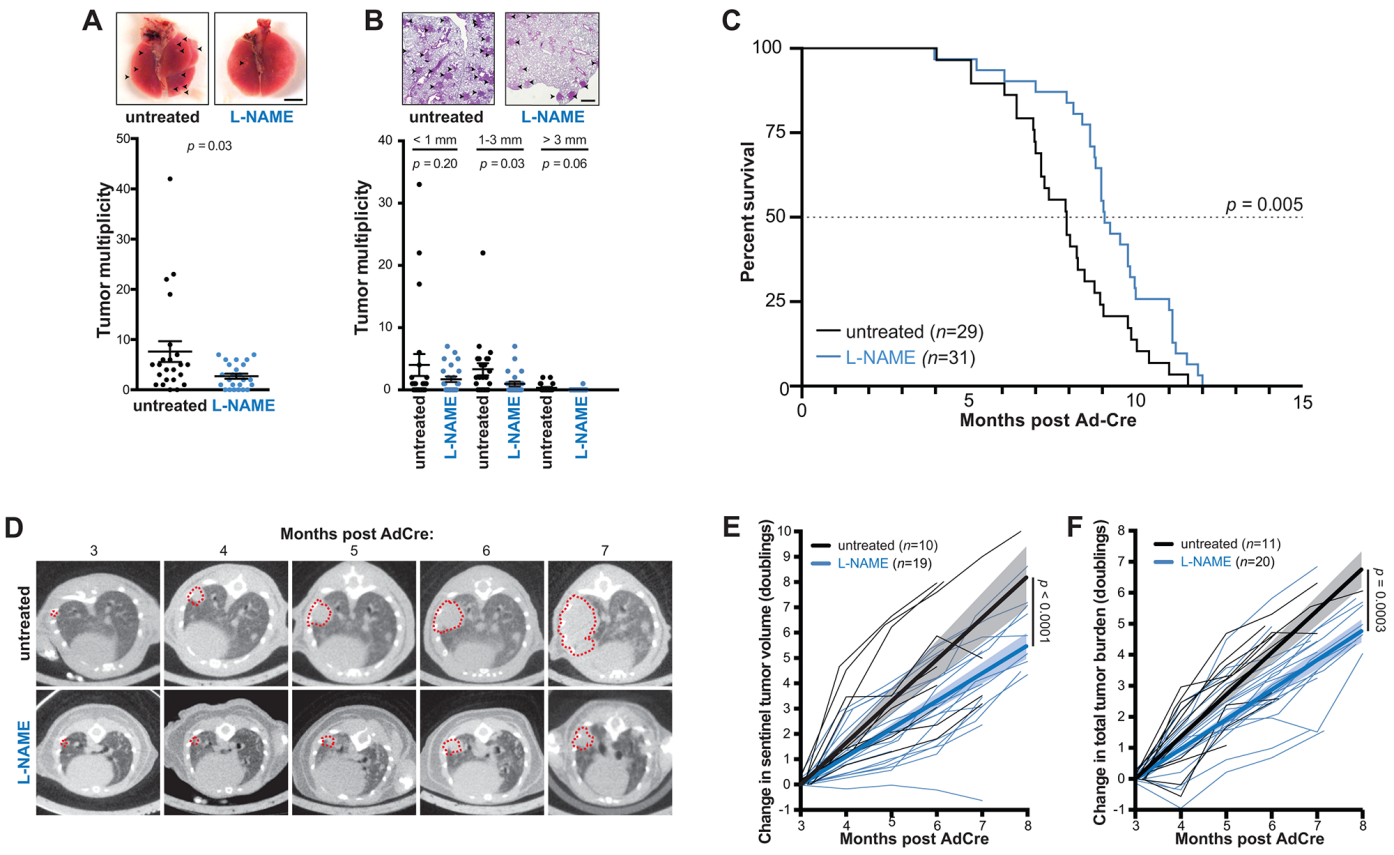
L-NAME treatment reduces lung tumor burden, inhibits lung tumor growth, and provides a survival benefit in mice developing *Kras* mutationpositive NSCLC **A.** *Kras^LSL-G12D/+^*;Trp53*^LSL-R172H/+^* mice administered AdCre to induce lung cancer were either untreated (*n*=24) or provided L-NAME in the drinking water ad libitum (*n*=23) until humanly euthanized four months later. Lungs were removed and tumors on the surface of the lungs (top panel, arrow: lesions, bar: 4 mm) quantitated and the mean ± SEM (bars) and number (closed circles) of lung tumors per mouse (tumor multiplicity) plotted (bottom panel). **B.** Lungs from the aforementioned mice were also sectioned, H&E stained, and the tumors detected by pathologic analysis (top panel, arrow: lesions, bar: 500 μm) quantitated based on the indicated sizes, and the mean ± SEM (bars) and number (closed circles) of these tumors per mouse (tumor multiplicity) plotted (bottom panel). **C.** Another cohort of *Kras^LSL-G12D/+^*;Trp53*^LSL-R172H/+^* mice were administered AdCre and either left untreated (*n*=29) or treated with L-NAME (*n*=31) as above until humanly euthanized upon reaching a moribundity endpoint, and the percent of mice surviving after AdCre administration versus time in months of both cohorts plotted. Dotted line: 50% survival. **D.** An example of CT-scans of an untreated (top) or L-NAME treated (bottom) mouse from the aforementioned cohorts at the indicated time points. Transverse planes with maximal tumor cross sectional area (denoted with a red dotted line) are shown. **E.** The change in segmented volume of a single sentinel tumor, or **F.** the sum of total lung tumor area (tumor burden), as assessed radiologically in individual mice over time from a subset of the above animals left untreated (black line, *n*=10 or 11 respectively) or treated with L-NAME (blue line, *n=*19 or 20, respectively). Thick lines and shading: linear regression ± 95% confidence intervals.

Mice heterozygous for the Cre-inducible oncogenic *Kras^LSL-G12D^* and dominant-negative *Trp53^LSL-R172H^* alleles were intranasally administered adenovirus encoding Cre recombinase (AdCre) to induce lung cancer [26]. Mice were then randomized to be untreated or provided L-NAME in the drinking water *ad libitum* (Supplementary Figure S1A) to achieve a dose sufficient to chronically inhibit NOS *in vivo* for upwards of 330 days [21]. Mice were euthanized four months later when disease is established (*e.g.* when at least one tumor is detected, see below), the lungs were removed and visually inspected for surface tumors, then sectioned and H&E stained for pathologic examination. This analysis revealed almost a three-fold reduction in visible tumors (Figure 1A) and a similar reduction in the number of tumors detected in H&E-stained lung sections (Figure 1B) in the L-NAME treated cohort. Thus, L-NAME treatment reduces tumor burden in a genetically engineered mouse model of *Kras* mutation-positive NSCLC.

### L-NAME therapy inhibits tumor growth and provides a survival benefit

To assess whether the drug L-NAME could extend survival, perhaps the most important indicator of clinical efficacy, the experiment was repeated as above, except mice were euthanized upon reaching a moribundity endpoint (Supplementary Figure S1A). Mice treated with L-NAME exhibited a 23% increase in median overall survival (OS), which amounted to 53 days (Figure 1C). A subset of these animals underwent monthly micro-computed tomography (CT) scans, which revealed slower tumor growth kinetics in the L-NAME treated cohort (Figure 1D). Specifically, there was a 33% reduction in the rate of doubling in volume of a single tumor (Figure 1E) and a 29% reduction in the rate of increase in the total lung tumor burden (Figure 1F) in the L-NAME treated mice. L-NAME has been argued to both enhance [27, 28] and suppress [20, 29] metastasis. However, the number of putative metastatic lesions at moribundity endpoint was similar between the untreated and L-NAME treated mice (Supplementary Figure S1B). In conclusion, L-NAME treatment impedes tumor growth and provides a survival benefit in a mouse model of *Kras* mutation-positive NSCLC.

### L-NAME therapy improves survival compared to carboplatin

**Figure 2:**
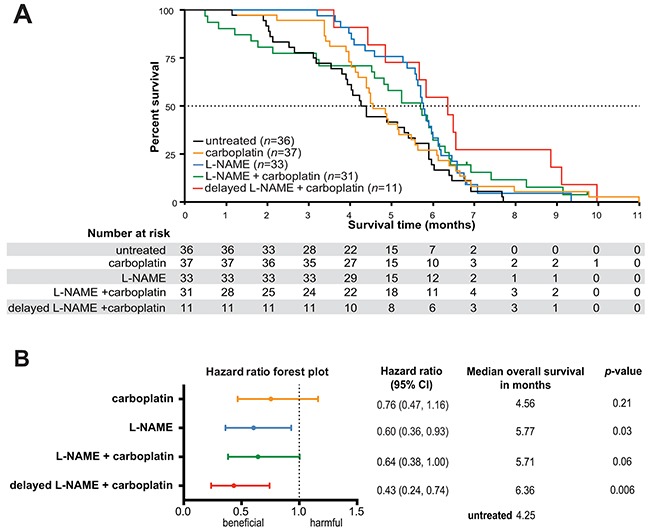
L-NAME treatment provides a survival advantage in mice with established *Kras* mutation-positive NSCLC, both as monotherapy and in combination with carboplatin **A.** *Kras^LSLG12D/+^*;Trp53*^LSL-R172H/+^* mice were administered AdCre to induce lung cancer and four months later randomized to the receive no treatment (*n*=36) or treatment with L-NAME (*n*=33), carboplatin (*n*=37), carboplatin and L-NAME *(n*=31), or carboplatin and 4-6 weeks later (delayed therapy), L-NAME (*n*=11). The percent survival versus time in months after treatments began was plotted for each cohort. Dotted line: 50% survival. **B.** Forest plot of hazard ratios and 95% confidence intervals for survival in aforementioned treatment groups in comparison to the untreated group. Dotted line: untreated group survival. *p*-values calculated for each treatment compared to untreated.

To rigorously evaluate the clinical potential of repurposing L-NAME for the treatment of *KRAS* mutation-positive NSCLC, *Kras*^*LSL-G12D/+*^;*Trp53*^*LSL-R172H/+*^ mice were administered AdCre as above. Four months later when frank disease is established (*e.g.* when at least one tumor is present), mice were randomized to receive no treatment, L-NAME as above, the platinum-based chemotherapeutic carboplatin once a day for five consecutive days, a regimen previously shown to recapitulate human tumor response in a similar mouse lung cancer model [25], or carboplatin as described, followed three days later after renal clearance of this drug [30] with L-NAME as above. Mice were euthanized upon reaching a moribundity endpoint. OS was defined as the time span between initiation of therapy and reaching endpoint, consistent with clinical practice (Supplementary Figure S1A). In agreement with the modest clinical therapeutic benefit of platinum-based chemotherapy in advanced NSCLC patients [31], mice treated with carboplatin exhibited a marginal but statistically insignificant improvement in the median OS (4.56 months) over the untreated group (4.25 months) that resulted in a hazard ratio (HR) of 0.76. In contrast, the L-NAME treatment group exhibited a clear improved median OS of 5.77 months and a HR of 0.60 over the untreated cohort, and trended towards a 27% increase in median OS over carboplatin chemotherapy (Figure 2A,B). L-NAME treatment thus appears to be more efficacious than the carboplatin regiment employed in the tested *Kras* mutation-positive NSCLC mouse model.

### L-NAME as maintenance therapy after carboplatin

L-NAME in combination with carboplatin trended towards an improved median OS of 5.71 months with a HR of 0.64 compared to the untreated mice (Figure 2A,B). Interestingly, the first quartile of this cohort reached a moribundity endpoint before the L-NAME treatment group (Figure 2A), suggestive of toxicity during the period that carboplatin and L-NAME were administered. Concurrent administration of erlotinib and carboplatin or taxanes has also been reported to be toxic unless dosed sequentially in a similar mouse model of lung cancer [25]. Given this, mice were randomized into an additional treatment arm consisting of carboplatin followed four to six weeks later by L-NAME. Not only did this delay eliminate the early moribundity, it also yielded the most significantly improved median OS of 6.36 months compared to the untreated cohort, with a HR of 0.43 (Figure 2A,B).

## DISCUSSION

Consistent with previous reports of a pro-tumorigenic role of NOS in lung cancer [6–9], we demonstrate that in a genetically engineered mouse model of *Kras*-driven NSCLC, L-NAME treatment inhibits lung tumor growth, reduces tumor burden, increases median OS, and improves HR, even when treatment is initiated in the presence of established disease. Although the mechanism of this anti-neoplastic activity remains to be elucidated, we note that none of the NOS isoforms were detected by RT-PCR in 11 of 12 cultures of enriched tumor cells isolated from the lung tumors induced by AdCre in *Kras^LSL-G12D/+^*;Trp53*^LSL-R172H/+^* mice (Supplementary Figure S1C), consistent with previous analysis of NOS expression in human lung cancer patients [10–14]. As such, we suggest that the target of L-NAME lies within the stromal compartment. While it remains formally possible that the target of L-NAME is something other than NOS, this drug is well established to inhibit NOS enzymes [32]. In agreement with the notion that the antineoplastic effect of L-NAME is linked to inhibition of NOS, we previously demonstrated that L-NAME elevated blood pressure in a similar *Kras^LSL-G12D/+^*;Trp53*^LSL-R172H/+^* mouse model of pancreatic cancer [21], validating NOS inhibition *in vivo* [33, 34]. Additionally, genetic ablating *iNOS* in a very similar oncogenic Kras-driven mouse lung cancer model phenocopies the effect of L-NAME, namely loss of iNOS led to a reduction in lung tumorigenesis [8]. We thus favor the interpretation that inhibition of one or more of the NOS isoforms in a stromal component accounts for the anti-neoplastic activity of L-NAME.

Treatment with carboplatin and shortly thereafter L-NAME trended towards an increase in the median OS and an improved HR compared to carboplatin alone. However, initiating L-NAME treatment four to six weeks after completion of carboplatin chemotherapy was far more effective, extending median OS by 50% compared to untreated mice, and trended towards an increase of 39% over carboplatin therapy. The underlying reason for the increased efficacy upon delaying L-NAME treatment remains to be elucidate. However, both platinum-based chemotherapeutics [35] and L-NAME [36] have nephrotoxic effects, and L-NAME has been reported to worsen the nephrotoxicity induced by the platinum-based drug cisplatin [37, 38]. In agreement, while no overt difference was detected in kidney function (*e.g.* elevated proteinuria, not shown), the average glomerular area was increased in mice treated with L-NAME and carboplatin compared to mice treated with carboplatin alone (Supplementary Figure S1D). These results suggest L-NAME may be best leveraged in an adjuvant setting after cessation of platinum-based chemotherapy.

As noted above, the delayed addition of L-NAME to carboplatin chemotherapy trended towards a 39% increase in median OS over carboplatin therapy. To put this into context, the combination of gemcitabine and cisplatin was found to increase OS by ~20% compared to cisplatin alone in a phase III NSCLC trial [39]. Given the current paucity of effective therapeutics for *KRAS* mutation-positive NSCLC, these observations support the evaluation of L-NAME or perhaps other NOS inhibitors for the treatment of this disease. As NO has been reported to suppress T-cell function [40], it is tempting to speculate that L-NAME may even find therapeutic value in conjunction with immune checkpoint inhibitors in the treatment of NSCLC. Lastly, as platinum-based drugs are widely used in the treatment of many types of solid tumors, NOS inhibitors may have use beyond the setting of NSCLC.

## MATERIALS AND METHODS

### Mice

For all studies, at two months of age, mixed background *Kras^LSL-G12D/+^*; Trp53*^LSL-R172H/+^* littermates (derived from strains 008179 and 008652, The Jackson Laboratory) were intranasally administered 6×10^6^ pfu AdCre (University of Iowa), as previously described [41]. Randomization for all studies was by cage for males and by mouse for females, mice were housed in a pathogen-free environment. All mouse care and experiments were approved by the Duke University IACUC and conducted in accordance with NIH guidelines for the care and use of laboratory animals.

### Preclinical trials described in Figure 1

In the studies corresponding to Figure 1, three days after AdCre administration mice were randomized to receive no treatment or provided drinking water supplemented with 1g/L of *N*^G^-nitro-L-arginine methyl ester (L-NAME, Sigma-Aldrich), as previously described [21]. Drinking water was changed with new drug three times a week. Treatment was initiated three days after adenovirus administration to limit effects of altering the acute inflammatory response to adenoviral infection [42]. Based on previous measurements of water consumption and average mouse weight, 1g/L of L-NAME taken *ad libitum* corresponds to an approximate dose of 180 mg/kg, which in mice is documented to inhibit eNOS activity yet can be dosed for extended periods of time [21]. Mice were then humanly euthanized at either a fixed time point (four months post AdCre) or upon reaching a moribundity endpoint. A subset of the latter mice were also radiologically imaged monthly using micro-computed tomography (CT) [43]. CT scans were reviewed in a blinded fashion using Image-J. Tumor volume was calculated by segmentation of serial transverse images of the most prominent lesion visualized at three months (initial scan) and anatomically correlated lesion in subsequent scans. Tumor burden was calculated by summing the largest cross-sectional area of all visualized lesions in transverse images. All data was normalized to individual tumor volume/burden at the time of initial scan to facilitate interpretation of relative tumor growth kinetics. Hence, mice without apparent tumor burden at three months were excluded from analysis. Data were plotted to reflect tumor doubling time (log2), and fitted with linear regression and 95% confidence interval as shown. Growth kinetics were compared using ordinary two-way ANOVA without matching. All statistical analyses and plots were done using PRISM6. Lungs were removed from euthanized mice in both experiments, and visible surface lesions quantitated. The lungs were then embedded in paraffin, sectioned at a thickness of 5 μm, and H&E stained. A minimum of five sections from five animals were examined, and representative images of tumor distribution are shown. Necropsies were performed on all euthanized animals and the presence of visible metastatic tumor lesions in the thoracic cavity, liver, kidneys and abdominal viscera was used to calculate the incidence (number per animal) of metastatic lesions in the two cohorts. Finally, lung tumors were microdissected from ten untreated mice and two from L-NAME treated mice and adapted to grow in culture as previously described [21]. RNA isolated from these 12 tumor enriched cultures, as well as heart tissue (eNOS-positive control), the macrophage cell line raw 264.7 (iNOS positive control), brain tissue (nNOS positive control) and the small intestine (negative control) was RT-PCR amplified with primers specific for *eNOS* (5′-CGATGTCACTATGGCAACCA and 5′-CCTGCAAAGAAAAGCTCTGG), *iNOS* (5′-CG TGAAGAAAACCCCTTGT and 5′-CGATGTCACA TGCAGCTTG), *nNOS* (5′-CTCGACCAATACTACTCCTCCATTAAGAGATTTGGC and 5′-CGCTGAACTCCAGGCCCCCAATCTCCAGCAGCATGTTGG), and control *eef1a* (5′-GGATTGCCACACGGCTCACATT and 5′-GGTGGATAGTCTGAGAAGCTCTC) mRNA and resolved by gel electrophoresis, similar to that previously described [21].

### Preclinical trial described in Figure 2

In the study corresponding to Figure 2, the experiment was performed exactly as described above, with the following three exceptions: First, all drug treatments were initiated four months after AdCre administration, the time that tumors were present in all animal analyzed by CT scanning in experiment corresponding to Figure 1. Second, mice were randomized to be *i*) untreated, *ii*) provided with drinking water supplemented with 1g/L of L-NAME, *iii*) injected intraperitoneally with carboplatin (Hospira) for a dose of 25 mg/kg once a day for five consecutive days, as previously described [25], and *iv*) treated with carboplatin above, then three days later when renal clearance of carboplatin was anticipated to be complete [30], mice were provided drinking water supplemented with 1g/L of L-NAME. Third, roughly half way through this preclinical trial a fifth treatment arm was added, namely mice were treated with carboplatin as above but then four to six weeks thereafter the mice were provided with drinking water supplemented with 1g/L of L-NAME. Mice were then humanly euthanized upon reaching a moribundity endpoint. Kidneys were also removed at endpoint from 4 mice treated with carboplatin and 5 mice treated with carboplatin and L-NAME, paraffin embedded, sectioned at a thickness of 5 μm and H&E stained. The area from 69 glomeruli from the first cohort and 87 from the second were measured and normalized to the average area from the carboplatin-treated cohort and reported as average area per glomeruli.

### Statistics

Statistical analyses were performed using GraphPad Prism software, version 6 (GraphPad Software) and Stata Statistical Software version 13.1 (StataCorp LP, College Station, TX). Comparisons between the groups were made using unpaired, 2-tailed *t*-tests with a 95% CI for continuous variables. OS was measured from time of AdCre administration (Figure 1C) or initiation of therapy (Figure 2) to endpoint. Survival analysis was assessed using Kaplan-Meier method, log-rank test, and Cox proportional hazards analysis. *p* value < 0.05 was considered statistically significant.

## SUPPLEMENTARY FIGURE


