# Supplementary material for: Treatment with the nitric oxide synthase inhibitor L-NAME provides a survival advantage in a mouse model of Kras mutation-positive, non-small cell lung cancer
Source: Oncotarget. 2016 Jun 7;7(27):42385–92. doi: 10.18632/oncotarget.9874 (PMC5173142; doi:10.18632/oncotarget.9874)
Supplement: Supplementary file 1 [file oncotarget-07-42385-s001.pdf]

SUPPLEMENTARY FIGURE

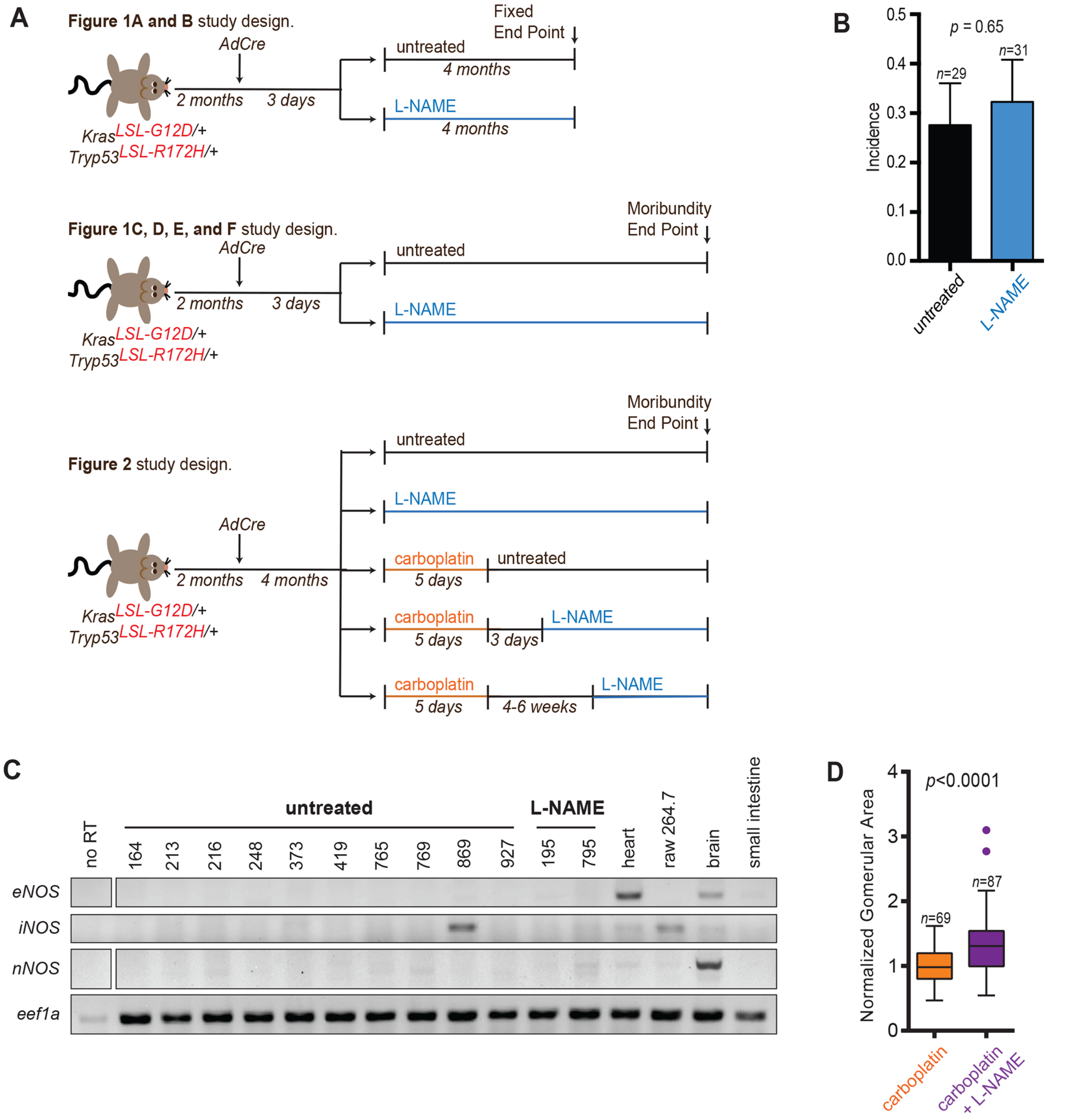

Supplementary Figure S1: A. Schematic of study design. **B.** Mean ± SEM incidence of visible metastatic lesions in untreated or L–NAME treated mice at morbundity endpoints. **C.** Semi-quantitative RT-PCR amplification of the indicated transcripts of cultures enriched in tumors cells derived from untreated or L–NAME treated mice. Heart and brain tissues, as well as murine macrophage cell line RAW 264.7 serve as positive controls for eNOS, nNOS, and iNOS expression, respectively. Small intestine is included as negative control. Representative of three independent experiments. **D.** Tukey box and whisker plot illustrating quantification of mean SEM glomerular area. 87 glomeruli from five mice in the L–NAME ad carboplating treated cohort and 69 glomeruli from four mice in the carboplatin only cohort were measured.
